# Supplementary material for: The rise and global spread of IMP carbapenemases (1996–2023): a genomic epidemiology study
Source: medRxiv. 2025 May 26:2025.05.25.25328332. Preprint. [Version 1] doi: 10.1101/2025.05.25.25328332 (PMC12148276; doi:10.1101/2025.05.25.25328332)
Supplement: Supplement 1 [file media-1.pdf]

Supplemental material for: The rise and global spread of IMP carbapenemases (1996-2023): a genomic epidemiology study

Authors

Ben Vezina[1+], Bhargava Reddy Morampalli[1+], Hoai-An Nguyen[1], Angela Gomez-Simmonds[2], Anton Y. Peleg[1,3,4], Nenad Macesic[*1,3,5]

[1] Department of Infectious Diseases, The Alfred Hospital and School of Translational Medicine, Monash University, Melbourne, Australia

[2] Division of Infectious Diseases, Department of Internal Medicine, UC Davis Health, Sacramento, California, USA

[3] Centre to Impact AMR, Monash University, Clayton, Australia

[4] Infection Program, Monash Biomedicine Discovery Institute, Department of Microbiology, Monash University, Clayton, Australia

[5] Infection Prevention & Healthcare Epidemiology, Alfred Health, Melbourne, Australia.

[+] These authors contributed equally

* Corresponding Author Dr. Nenad Macesic, Department of Infectious Diseases, The Alfred Hospital and School of Translational Medicine, Monash University, Level 1 Alfred Lane House, Alfred Hospital, 55 Commercial Rd, Melbourne, VIC 3004, Australia. Email: nenad.macesic1@monash.edu

Supplemental Figures

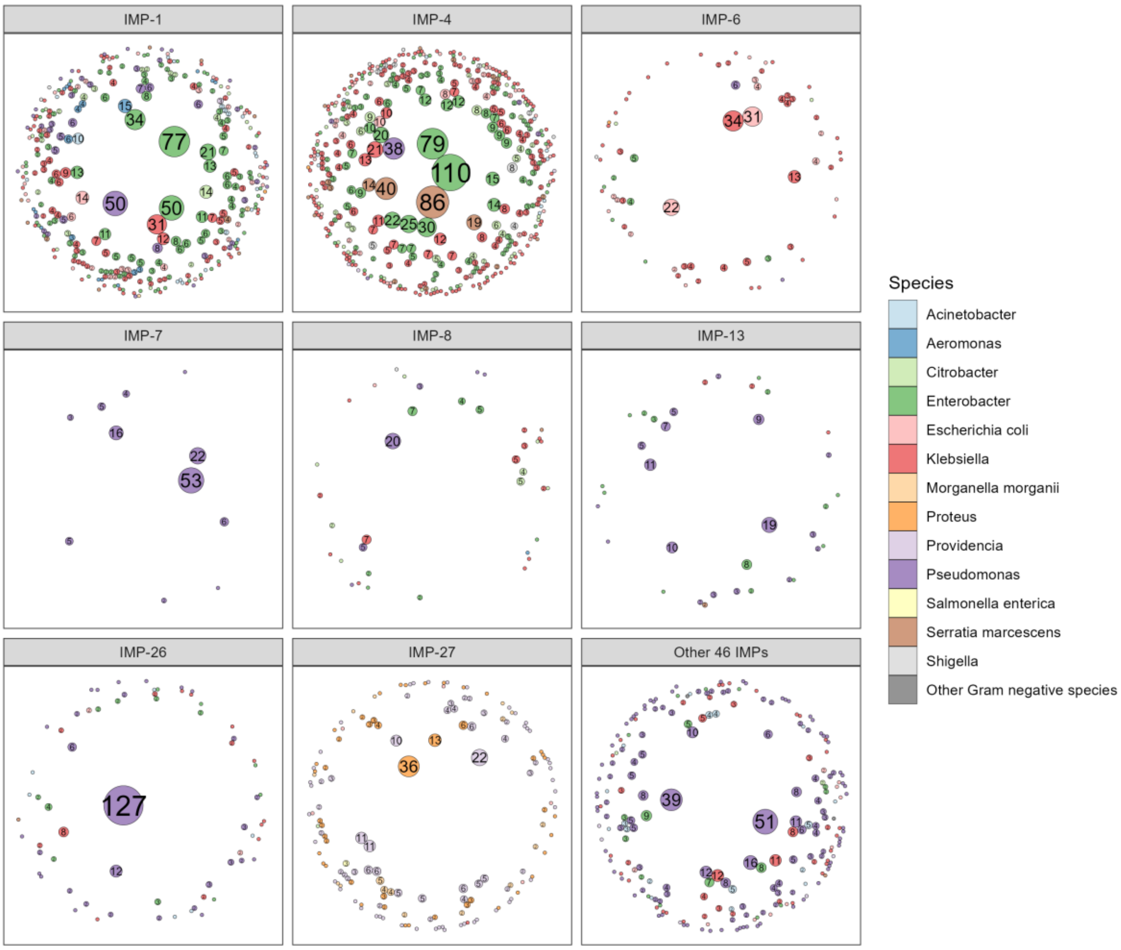

Fig. S1: Size of IMP-clusters within dataset. Number of genomes within IMP-cluster shown by size of bubble and text. No text is shown for IMP-clusters with n=1 genome.

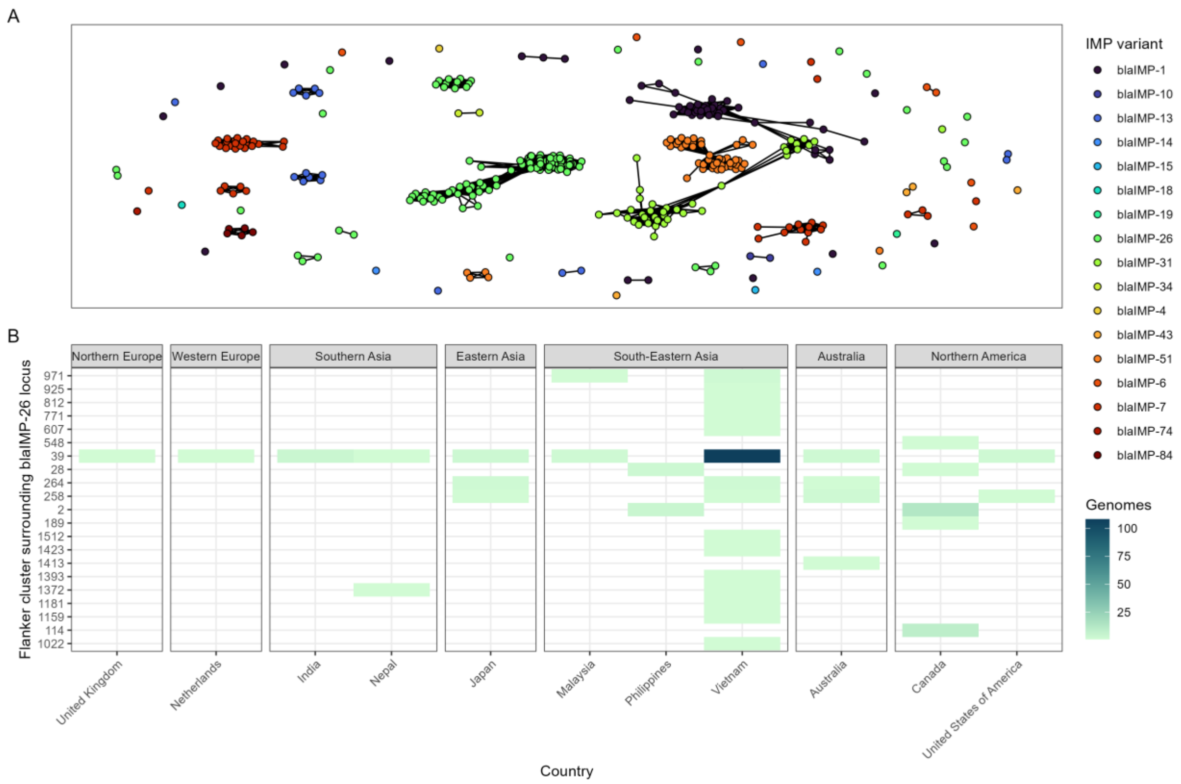

Fig. S2: *bla*$_{IMP}$-carrying *P. aeruginosa* ST235 has global and diverse spread **A:** Network of clonally-linked *P. aeruginosa* ST235 genomes, coloured by *bla*$_{IMP}$ variant. **B:** Heatmap of IMP-26-containing *P. aeruginosa* ST235 showing regional-specific independent acquisition of the IMP-26 gene.

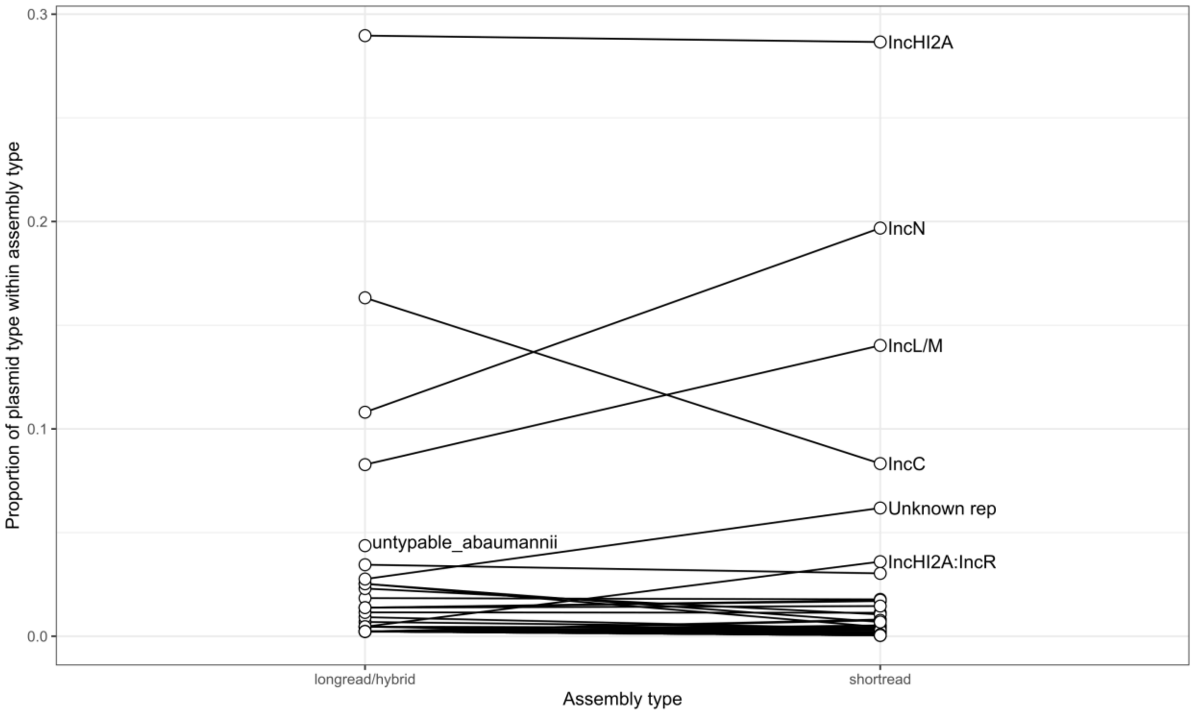

Fig. S3: Proportion of plasmid clusters in long-read/hybrid dataset vs full dataset including short read draft assemblies.

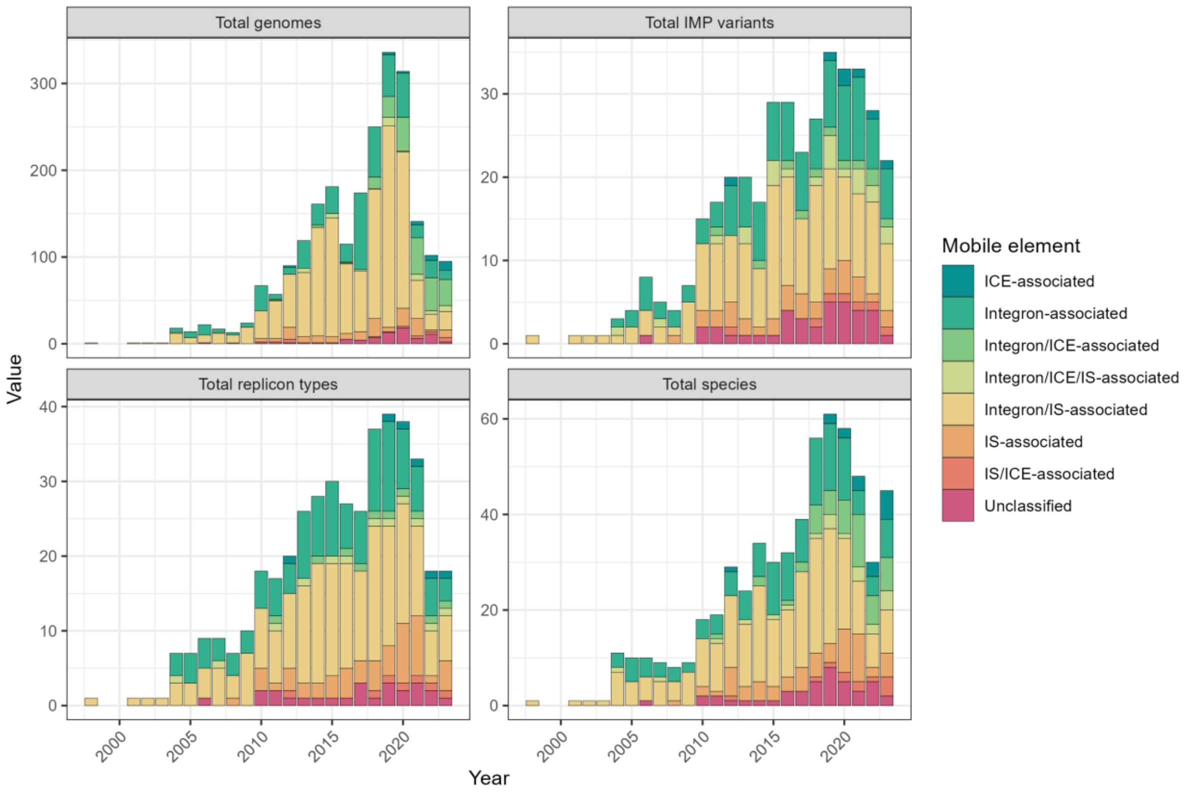

Fig. S4: Mobile elements have been associated with IMP carbapenemases over time Cumulative column graph showing the breakdown of associated mobile elements and their spread across numbers of species, *bla*$_{IMP}$ variants, total genomes and plasmid types. Raw data found in Table S1.

Supplemental Table legends

Table S1: Table of all data used in this study, including accession numbers, analysis results and metadata.

Table S2: *bla*$_{IMP}$ variants over time, supporting information for Fig. 1.

Table S3: *bla*$_{IMP-26}$ and *bla*$_{IMP-27}$ supporting information for Fig. 3.

Table S4: *bla*$_{IMP}$ variants and their global distributions

Table S5: Association between bacterial lineages and *bla*$_{IMP}$ variants

Table S6: IMP-clusters and their global distribution and makeup

Table S7: Plasmid clusters and their spread across multiple countries and geographical regions for long read only genomes

Table S8: 'Propagator' strain-plasmid pairings and 'connector' strains.

Table S9: Summary of isolation sources and genome counts, supporting information for Fig. 7
